# Exercise Effects on Autonomic Nervous System Activity in Type 2 Diabetes Mellitus Patients over Time: A Meta-Regression Study

**DOI:** 10.3390/healthcare12121236

**Published:** 2024-06-20

**Authors:** Jui-Kun Chiang, Po-Chen Chiang, Hsueh-Hsin Kao, Weir-Chiang You, Yee-Hsin Kao

**Affiliations:** 1 Department of Family Medicine, Dalin Tzu Chi Hospital, Buddhist Tzu Chi Medical Foundation, No. 2, Minsheng Road, Dalin, Chiayi 622, Taiwan; roma@tzuchi.com.tw; 2 Department of Medicine, College of Medicine, National Taiwan University, Taipei 100, Taiwan; b08401094@ntu.edu.tw; 3 Department of Radiation Oncology, Taichung Veterans General Hospital, Taichung 407, Taiwan; kaogrady8176@gmail.com; 4 Department of Family Medicine, Tainan Municipal Hospital (Managed by Show Chwan Medical Care Corporation), 670 Chung-Te Road, Tainan 701, Taiwan

**Keywords:** exercises, autonomic nervous system, heart rate variability (HRV), type 2 diabetes mellitus (T2DM)

## Abstract

Background: Diabetic autonomic neuropathy is a common complication of type 2 diabetes mellitus (T2DM), especially in patients with long-term, poorly controlled diabetes. This study investigates the effects of exercise on autonomic nervous system activity in T2DM patients over time. Methods: A literature review using MEDLINE, Embase, Cochrane Library, Scopus, and PubMed identified studies assessed via heart rate variability. Papers were categorized into three groups: immediate effects (within 60 min), short-term effects (2–3 months), and long-term effects (over 4 months). Results: Nine articles with 161 T2DM patients were included in the meta-analysis. RMSSD changes after exercise were −4.3 (*p* = 0.227), 8.14 (*p* < 0.001), and 4.17 (*p* = 0.002) for the immediate, short-term, and long-term groups, respectively. LF/HF ratio changes were 0.21 (*p* = 0.264), −3.04 (*p* = 0.102), and −0.05 (*p* = 0.006) for the respective groups. Meta-regression indicated age, male gender, and exercise duration were associated with increased RMSSD, with coefficients of 2.36 (*p* = 0.001), 13.76 (*p* = 0.008), and 1.50 (*p* = 0.007), respectively. Age positively correlated with the LF/HF ratio, with a coefficient of 0.049 (*p* = 0.048). Conclusions: Regular exercise (≥3 times per week) for over 2 months increases parasympathetic activity in T2DM patients, while sympathetic activity decreases significantly after 4 months. Further study is needed to validate these findings.

## 1. Introduction

In 2021, more than five million people worldwide were living with diabetes [1]. Diabetes remains a significant public health challenge, with cardiovascular disease (CVD) representing the primary burden of type 2 diabetes mellitus (T2DM) in terms of morbidity, mortality, and socioeconomic costs for individuals and societies [2].

Diabetic autonomic neuropathy (DAN), characterized by impaired function of peripheral autonomic nerves, presents a broad array of manifestations affecting various organ systems, including cardiovascular, gastrointestinal, genitourinary, sudomotor, vasomotor, and neuroendocrine systems, and is prevalent among individuals with diabetes mellitus. The prevalence of DAN rises with the duration of diabetes. It may be detected in up to 7 percent of patients at the time of diabetes diagnosis [3,4]. Cardiac autonomic neuropathy (CAN) is another serious complication of T2DM that is strongly associated with approximately a five-fold increased risk of cardiovascular mortality. Clinical correlates or risk markers for CAN include age, duration of DM, glycemic control, hypertension, dyslipidemia, and the development of other microvascular complications [5].

Various measurements are available to assess cardiac autonomic neuropathy in patients with type 2 diabetes mellitus (T2DM). However, the standard cardiovascular reflex tests remain the gold standard for the assessment of cardiac autonomic neuropathy [6]. The heart rate variability (HRV) method is a non-invasive, reliable, and pain-free measurement to assess autonomic nervous activity [7]. Given the significant divergence in study methodologies, standardized tools are evidently needed to enhance the quality of measurements in current meta-analyses. Resting HRV is influenced by both psychological factors [8] and physiological factors, including age, gender, body mass index, chronic conditions [9], heart function, and heart diseases [10]. The root mean square of successive heart beat interval differences (RMSSD) for parasympathetic activity is endorsed by the Task Force of the European Society of Cardiology and the North American Society of Pacing and Electrophysiology [9] as well as by Sequeira et al. [11] and Kobayashi et al. [12]. Additionally, the ratio between the absolute power of the high-frequency (HF) band (0.015–0.400 Hz) and the absolute power of the low-frequency (LF) band (0.040–0.150 Hz), referred to as the LF/HF ratio, has been identified as an indicator of sympathovagal balance, which indirectly reflects the degree of cardiac sympathetic nervous system activity. [13]. In our current study, we employed the LF/HF ratio, a frequency-domain measurement index, as a proxy for sympathetic activity, following the methodology outlined in previous studies by AlQatari et al. [14] and Kobayashi et al. [15].

A systematic review reported that T2DM was associated with a decrease in parasympathetic activity, as indicated by RMSSD, while findings regarding sympathetic activity, as indicated by the LF/HF ratio, were inconclusive [7]. A previous systematic review reported that exercise, encompassing aerobic exercise combined with resistance training, high-intensity interval training, and progressive resistance training, had a positive impact on cardiac autonomic function in individuals with T2DM [16]. Additionally, a previous systematic meta-analysis reported a lower RMSSD but no change in the LF/HF ratio in T2DM patients compared to healthy participants [7]. Another systemic meta-analysis reported that exercise training, especially after endurance training and supervised training, improved HRV parameters in T2DM patients [17]. However, discussion on the duration of exercise training and its impact on autonomic activity improvement in T2DM patients is limited. Therefore, the aim of the present study is to investigate the effects of exercise on autonomic nervous system activity in patients with T2DM over a specified period of time.

## 2. Materials and Methods

### 2.1. Study Design

A systematic review and meta-analyses were conducted to identify studies examining the effects of exercise on autonomic nervous system activity in patients with type 2 diabetes mellitus over time. The Preferred Reporting Items for Systematic Reviews and Meta-Analyses (PRISMA) guidelines were followed [18]. The study protocol was reviewed and approved by the Research Ethics Committee of the Buddhist Dalin Tzu Chi Hospital in Taiwan (No. B11301022). The study was registered with PROSPERO(CRD42024552655).

### 2.2. Search Strategy

**Figure 1:**
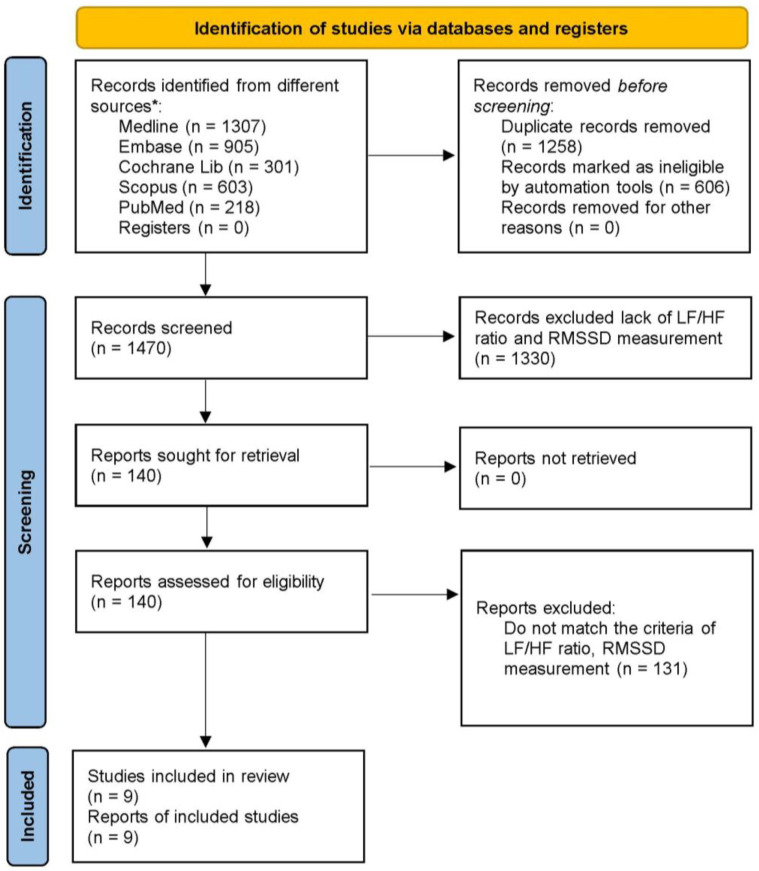
PRISMA flow diagram of the study selection. * Each database along with the corresponding numbers of records identified.

A wide search of the literature was conducted for studies indexed on MEDLINE, Embase, Cochrane Library, Scopus, and PubMed from their inception to 19 April 2024 in English (Figure 1). In our current study, we included clinical trials, case-controlled studies, and observational studies while excluding case-reported studies, poster articles, retrospective studies, systematic reviews, and meta-analysis studies. We utilized terms like “exercise”, including variations such as “run*”, “walk*”, “jog*”, “treadmill*”, “tread mill*”, and “racewalk*”. We searched for these terms within the title, abstract, and keywords. We systematically searched Ovid Medline, Embase, Cochrane Library, PubMed, and Scopus (Google Scholar) for relevant articles.

### 2.3. Study Selection

We applied the PICOS (participants, interventions, comparisons, outcomes, and study design) model to define the research question. The inclusion criteria were as follows: (1) patients with T2DM, (2) receiving regular exercise, and (3) studies in which the methods used to assess parasympathetic activity (such as RMSSD) and evaluate sympathetic activity (such as the LF/HF ratio) were presented as mean ± SD during the data extraction process. Studies were excluded if the data were ambiguous, no data after exercise were provided, or communication with the corresponding authors was not possible. The extracted data were verified by two independent reviewers (Y.-H.K. and J.-K.C.), which were further revalidated by H.-H.K. Any disagreements between the reviewers were resolved through consultation with a statistician. Subsequently, these two authors further screened the papers and selected nine for analysis.

### 2.4. Data Extraction

The following data from the included studies were recorded: author, year of publication, sample size, age (years), gender (percentage of women), presence of type 2 diabetes mellitus, and the values of parasympathetic activity (such as RMSSD) and sympathetic activity (such as the LF/HF ratio), which were presented as mean ± SD. The data extraction was independently conducted by two independent reviewers (Y.-H.K. and J.-K.C.). A standardized form for data extraction is provided in Supplementary Table S1.

### 2.5. Methodological Quality of Included Studies

After obtaining the eligible articles, data extraction and methodological quality assessment were carried out by two independent reviewers (Y.-H.K. and J.-K.C.). Methodological quality assessment was evaluated using the Downs and Black checklist [19]. This tool consists of 27 items, including five subscales, which are as follows: reporting, external validity, internal validity (study bias and confounding), selection bias, and study power. Poor quality is considered when a score of 14 or less is achieved, fair quality between 15 and 19, good quality between 20 and 25, and excellent quality when the score is higher or equal to 26 [20].

### 2.6. Data Collection and Analysis

The data were extracted into a standardized form by these two authors and then extracted into summary of findings tables. The authors were contacted to seek clarification on any ambiguous data regarding the methods used to assess parasympathetic activity, such as RMSSD, and evaluate sympathetic activity, such as detecting the LF/HF ratio, during the data extraction process. The studies, published between 2003 and 2024, included five randomized designs [21,22,23,24,25].

### 2.7. Data Synthesis and Publication Bias Check

After extracting and critically appraising the data, the included papers were analyzed using narrative synthesis. To guide the narrative synthesis process, we consulted the flowchart by Rodgers et al. [26], which offers guidance on synthesizing data, identifying patterns, assessing the strength of these patterns, and drawing conclusions based on them. To identify potential publication bias, we performed a funnel plot analysis. Egger’s test was particularly used to assess the symmetry of the funnel plot for detecting publication bias. The articles were screened, and discrepancies were resolved by two independent reviewers (Y.-H.K. and J.-K.C.). J.-K.C. also served as the statistician for the current study.

### 2.8. Statistical Analysis

The meta-analysis utilized the Knapp and Hartung adjustments to compute the weighted average of the studies, comparing pre- and post-exercise measurements across various intervals. The pooled summary effect was illustrated in a forest plot. Heterogeneity among the collected studies was assessed using the chi-square Q test and the I^2^ statistics. In cases of substantial heterogeneity, random-effects models were applied. Subgroup analyses for mean differences were conducted to explore heterogeneity, and meta-regression was employed to identify significant moderators.

To ensure the analysis’s quality, basic model fitting techniques, including goodness-of-fit (GOF) assessment and regression diagnostics, were applied in the meta-regression analysis for mean differences. The coefficient of determination, R^2^, was reported to evaluate the GOF of the fitted linear meta-regression model. Additionally, statistical tools for regression diagnostics were used to examine the data. The publication bias, residual analysis, and identification of influential studies were examined. Trial sequential analysis (TSA) was employed to determine if the sample size was adequate, relying on a type I error rate of 5% and a power of 90%. This method delineates boundaries for benefit, harm, and the inner wedge. Additionally, it evaluates overall efficacy through cumulative z-scores [27].

All statistical calculations were performed using R statistical software, version 4.2.3 (R Foundation for Statistical Computing, Vienna, Austria). A two-sided *p*-value ≤ 0.05 was considered statistically significant.

## 3. Results

We identified 1307 papers in the Ovid Medline database, 905 papers in the Embase database, 301 papers in the Cochrane Library database, 603 papers in the Scopus database, and 218 papers in the PubMed database. In total, we found 3334 papers. Among these, 1258 studies were excluded due to duplication, and 606 studies were excluded for not meeting the PICO criteria. Subsequently, we included 140 papers that specifically addressed T2DM. Additionally, 131 full-text articles were excluded because they did not meet the criteria for measuring autonomic nervous system activity, such as the presentation of median values or logarithmic values of the LF/HF ratio or RMSSD. Finally, nine studies involving 161 T2DM patients were included for meta-analysis. The methodological quality of these studies was evaluated using the Downs and Black quality assessment method, and all articles were classified as fair. These nine studies provided data on 161 T2DM patients (mean age, 54.97 years; 39.6% women), and details of these nine studies are presented in Table 1. These articles comprised both experimental and control groups, incorporating various exercise modalities such as aerobic exercise, resistance exercise, cycling, and self-paced walking on a treadmill. For the influence of exercise duration on T2DM, we classified these nine studies into three groups: immediate effects of exercise within 60 min, short-term effects spanning 2–3 months, and long-term effects exceeding 4 months. Four studies, comprising nine subgroups, were categorized under the immediate group [21,22,28,29]; two studies, with four subgroups, were classified under the short-term group [23,24]; and three studies, representing four subgroups, were allocated to the long-term group [25,30,31].

**Figure 2:**
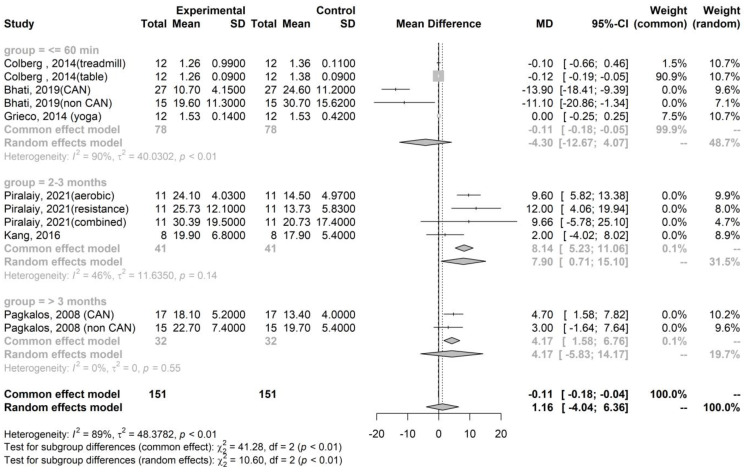
Summary of the meta-analysis on the effect of training exercises on RMSSD in T2DM [22,23,24,28,29,31].

**Figure 3:**
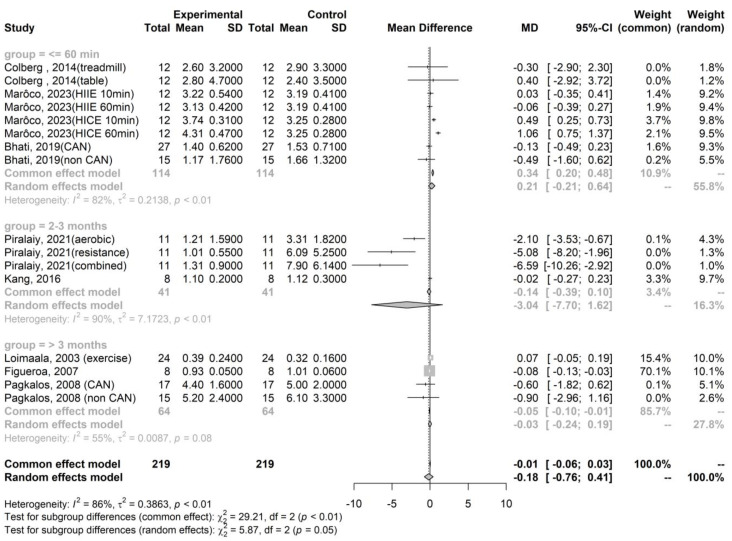
Summary of meta-analysis on the effect of training exercise on the LF/HF ratio in T2DM [21,22,23,24,25,28,30,31].

We observed that the mean difference in parasympathetic activity after exercise, as indicated by RMSSD, compared to pre-exercise levels, was −4.3 (95% confidence interval [CI]: −12.67–4.07, *p* = 0.227), 8.14 (95% CI: 5.23–11.06, *p* < 0.001), and 4.17 (95% CI: 1.58–6.76, *p* = 0.002) in the immediate (within 60 min), short-term (2–3 months), and long-term (4 or more months) groups, respectively (Figure 2). Similarly, the mean difference in sympathetic activity after exercise, represented by the LF/HF ratio, compared to pre-exercise levels, was 0.21 (95% CI: −0.21–0.64, *p* = 0.264), −3.04 (95% CI: −7.70–1.62, *p* = 0.102), and −0.05 (95% CI: −0.10–−0.01, *p* = 0.006) in the immediate, short-term, and long-term groups, respectively (Figure 3).

In our present study, we observed that the types of exercise in the short-term group included aerobic exercise (one subgroup), resistance training (one subgroup), or a combination of both (two subgroups). For the long-term group, the types of exercise comprised endurance training (one subgroup), walking training (one subgroup), and aerobic exercise (two subgroups). In the immediate-term group, the types of exercise included walking on a treadmill (one subgroup), table tennis (one subgroup), yoga (one subgroup), a maximal exercise test (two subgroups), moderate-intensity continuous exercise (MICE) (two subgroups), and acute high-intensity interval exercise (HIIE) (two subgroups).

**Figure 4:**
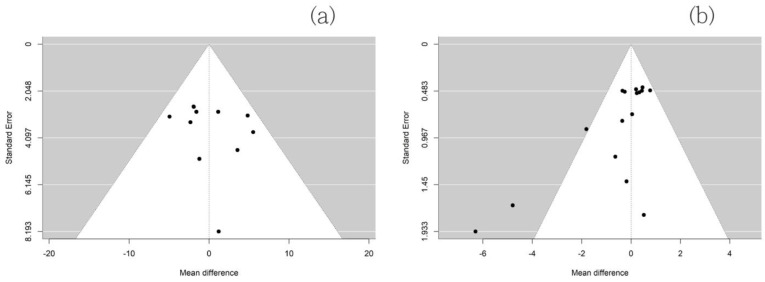
The funnel plots for (**a**) RMSSD and (**b**) LF/HF ratio. Each included study is represented as a black dot.

**Figure 5:**
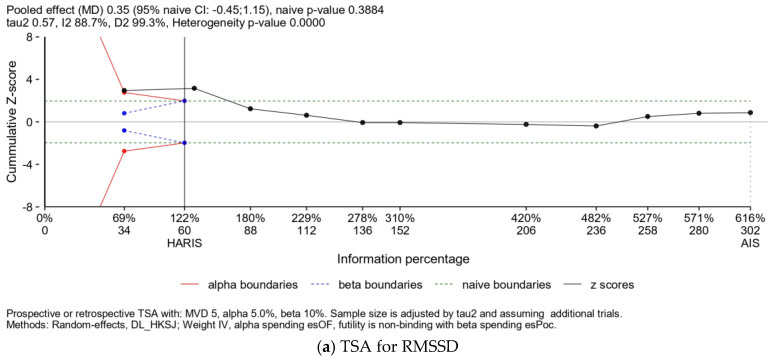

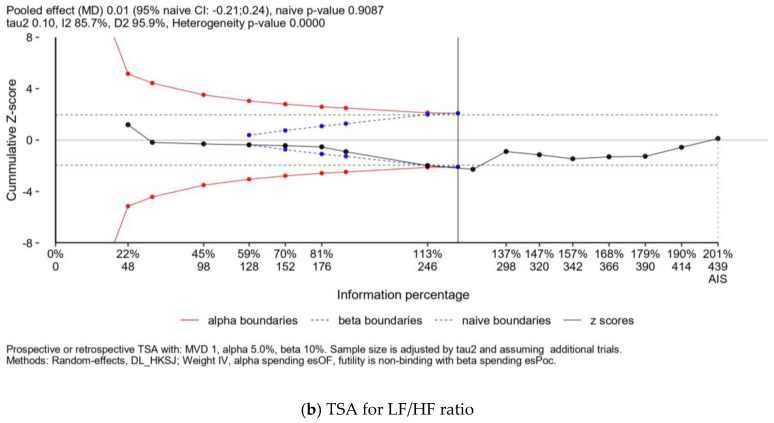
Trial sequential analysis for (**a**) RMSSD and (**b**) LF/HF ratio.

Finally, as illustrated in Table 2, we conducted a mixed-effects linear meta-regression analysis using data from these nine studies to identify predictors for RMSSD and the LF/HF ratio. The meta-regression model indicated that age, male gender, and duration of exercise were associated with increased RMSSD after exercise. The estimated coefficients for these factors were 2.36 (95% CI: 1.01–3.72, *p* = 0.001), 13.76 (95% CI: 3.59–23.94, *p* = 0.008), and 1.50 (95% CI: 0.41–2.59, *p* = 0.007), respectively. Egger’s test did not detect funnel plot asymmetry for RMSSD (*p* = 0.489). Additionally, an R-square value of 0.836 indicated an excellent fit. The meta-regression model revealed that age was significantly associated with an increased LF/HF ratio after exercise, with an estimated coefficient of 0.05 (95% CI: 0.001–0.098, *p* = 0.048). Eggers’ test does not indicate the presence of funnel plot asymmetry for the LF/HF ratio (*p* = 0.250). However, the R-squared value was 0.093 (Figure 4). In the post-hoc analysis using trial sequential analysis, the cumulative Z-line crossed the boundary for the required sample size at the 95% confidence level for both RMSSD and the LF/HF ratio. Thus, it seems that the amount of data gathered in this study should be sufficient to explore this topic adequately. In the current TSA, we set alpha (type I error) at 0.05 and beta (type II error) at 0.1 (power at 0.9). Additionally, Tau^2^ was obtained at 0.10 for LF/HF and 0.57 for RMSSD (Figure 5). Table 3 contains details of the types of exercise used in each study and group, emphasizing the differences between the exercise methods and their potential influence on autonomic nervous system activity.

We further analyzed the data after excluding the immediate group and observed that the pooling RMSSD and LF/HF ratio after exercise, compared to pre-exercise levels, was 5.92 (95% CI: 3.98–7.86, *p* < 0.001) and −1.34 (95% CI: −3.14–0.46, *p* = 0.123), respectively. The trial sequential analysis (TSA)-adjusted 95% CI for the LF/HF ratio was −0.43 to 0.08. In the post-hoc analysis using TSA for the LF/HF ratio, the cumulative Z-line did not cross the boundary for benefit and fell within the futility boundary. Therefore, further studies on the benefits of exercise duration exceeding two months in T2DM patients might not be necessary for the LF/HF ratio. For RMSSD, the TSA-adjusted 95% CI was 3.98 to 7.86, and the cumulative Z-line crossed the trial monitoring boundary for benefit. However, this analysis for RMSSD did not reach the ideal sample size. Consequently, further large-scale studies on the benefits of exercise duration exceeding two months in T2DM patients are warranted for RMSSD.

## 4. Discussion

In the present meta-analysis, we observed that parasympathetic activity, as indicated by RMSSD, decreased immediately following exercise (within 60 min) but increased over the short term (2–3 months) and long term (4 months or more) with regular exercise (≥3 times per week) in patients with T2DM. However, the decrease in sympathetic activity after exercise, as indicated by the LF/HF ratio, was significant only in the long-term exercise group (4 months or more).

A prior study suggested that exercise therapy may improve HRV in patients with myocardial infarction, chronic heart failure, and those undergoing revascularization by increasing vagal tone and decreasing sympathetic activity. One hypothesis is that a shift toward greater vagal modulation, possibly mediated by angiotensin II and nitric oxide, may positively affect the prognosis of these individuals [32]. However, in the current study, sympathetic activity, as indicated by the LF/HF ratio, did not decrease in the immediate and short-term exercise groups but significantly decreased in the long-term group (exercise duration ≥4 months in individuals with T2DM). A previous study reported that exercise training could enhance parasympathetic activity, subsequently reducing the risk of lethal arrhythmias. The mechanism may involve the mediation of angiotensin II and nitric oxide in the effect of exercise on improving parasympathetic activity [32]. A previous study reported that regular exercise, particularly endurance training over a period of about three months, increased sympathetic and parasympathetic activity, as indicated by LF and HF measurements, in individuals with T2DM who had no or early CAN. However, this benefit was not observed in individuals with T2DM who had definite or severe CAN [33]. This may be due to a high genetic risk in this subpopulation, influenced significantly by gene–environment interactions, where intense physical activity is associated with an increased risk of cardiovascular outcomes [34].

A previous systematic meta-analysis reported that patients with type 2 diabetes mellitus (T2DM) had a lower RMSSD but no change in the LF/HF ratio compared to healthy participants [7]. Another systematic meta-analysis found that exercise training, especially endurance training and supervised training, could increase RMSSD and decrease the LF/HF ratio in T2DM patients [17]. However, the study by Picard et al. had a small number of participants and did not find a significant association between the duration of exercise and improvements in HRV. In the current study, parasympathetic activity, as indicated by RMSSD, increased after more than 2 months of regular exercise. Additionally, sympathetic activity, as indicated by the LF/HF ratio, significantly decreased after more than 4 months of regular exercise among individuals with T2DM.

The benefits of exercise training may indeed vary depending on the characteristics of the patients. A separate systematic review indicated that exercise effects were influenced by age (age <50), male gender, and individuals with T2DM, who appeared to experience greater benefits [35]. However, another systematic review found that age and gender were not associated with improvements in HRV parameters in T2DM patients [17]. In the current study, we observed a significant positive association between male gender, age, and duration of exercise and a decrease in parasympathetic activity in individuals with T2DM. TSA was conducted in the current study. We set alpha (type I error) at 0.05 and beta (type II error) at 0.1 (power 0.9). The number of collected studies exceeded the required information size [36], suggesting that the amount of data gathered should be sufficient to explore this topic adequately. Thus, it seems that the data collected in this study should be sufficient to explore this topic comprehensively.

There are several limitations to the current study. Firstly, we acknowledge the inherent limitations of all meta-analyses [37], including the constraints and biases present in the individual studies analyzed. Because the studies we collected followed the guidelines of the Preferred Reporting Items for Systematic Reviews and Meta-Analyses (PRISMA), selection bias might be limited. However, a potential limitation of the current study is that we did not use search terms related to physical activity, physical training, tai chi, or other exercises. Secondly, we conducted the meta-analyses solely on published articles, thereby potentially exposing them to publication bias. However, the use of broader keywords in our search strategy likely reduced the number of missing studies. Additionally, the included studies were homogeneous according to funnel plots, and the populations investigated in the current study appeared to be equally distributed around the world. Thirdly, there is substantial methodological variation in the literature regarding the responses of heart rate variability (HRV) to exercise, including exercise protocols and HRV analysis techniques. This warrants further research to standardize methods and ensure consistency in exercise protocols. Lastly, the low R-squared value of 0.093 for the LF/HF ratio in the current meta-regression study suggests that the sample size might have been insufficient. Further studies are warranted to elucidate these findings.

## 5. Conclusions

In the current meta-analysis, we found that parasympathetic activity, as indicated by RMSSD, decreased immediately following exercise but increased after regular exercise (≥3 times per week) for more than 2 months in patients with T2DM. However, the decrease in sympathetic activity following exercise, as evidenced by the LF/HF ratio, became significant after engaging in exercise for 4 months or longer. In the post-hoc analysis using the TSA, the cumulative Z-lines finally did not cross the boundary for benefit and fell in the futility boundary for both RMSSD and the LF/HF ratio. Further study is needed to validate these findings.

In the current meta-regression study, we found that age, male gender, and duration of exercise were associated with increased RMSSD after exercise. The meta-regression model revealed a significantly positive association between age and the LF/HF ratio after exercise.

## Figures and Tables

**Table 1:** Characteristics of studies included in this systematic review.

Publication	Kinds of Exercise	Health Statuses	N, Age (Years Old)	Sympathetic Activity, Measured by the LF/HF Ratio	Parasympathetic Activity, Measured by the RMSSD
Colberg, 2014 [28]	Walk on a treadmill	Uncomplicated T2DM	N = 12, 58.7 ± 2.4	Pre-exercise: 2.9 ± 3.3 Immediate post-exercise: 2.6 ± 3.2	Pre-exercise: 1.4 ± 0.1 Immediate post-exercise: 1.3 ± 1.0
	Table tennis	Uncomplicated T2DM	N = 12, 58.7 ± 2.4	Pre-exercise: 2.4 ± 3.5 Immediate post-exercise: 2.8 ± 4.7	Pre-exercise: 1.4 ± 0.1 Immediate post-exercise: 1.3 ± 0.1
Marôco, 2023 [21]	HIIE 10min	T2DM	N = 12, 67 ± 8	Pre-exercise: 3.2 ± 0.4 Immediate post-exercise: 3.2 ± 0.5	Not available
	HIIE 60min	T2DM	N = 12, 67 ± 8	Pre-exercise: 3.2 ± 0.4 Immediate post-exercise: 3.1 ± 0.4	Not available
	MICE 10min	T2DM	N = 12, 67 ± 8	Pre-exercise: 3.3 ± 0.3 Immediate post-exercise: 3.7 ± 0.3	Not available
	MICE 60min	T2DM	N = 12, 67 ± 8	Pre-exercise: 3.3 ± 0.3 Immediate post-exercise: 4.3 ± 0.5	Not available
Bhati, 2019 [23]	A maximal exercise test	T2DM with CAN	N = 27, 51.7 ± 6.1	Pre-exercise: 1.5 ± 0.7 Immediate post-exercise: 1.4 ± 0.6	Pre-exercise: 24.6 ± 11.2 Immediate post-exercise: 10.7 ± 4.2
	A maximal exercise test	T2DM without CAN	N = 15, 50.2 ± 6.4	Pre-exercise: 1.7 ± 1.3 Immediate post-exercise: 1.2 ± 1.8	Pre-exercise: 30.7 ± 15.6 Immediate post-exercise: 19.6 ± 11.3
Grieco, 2014 [29]	Yoga	T2DM	N = 12, 54.9 ± 7.4	Not available	Pre-exercise: 1.5 ± 0.4 Immediate post-exercise: 1.5 ± 0.1
Piralaiy, 2021 [23]	Aerobic training (25–45 min per day, 3–5 times per week for 12 weeks)	T2DM	N = 11, 55.2 ± 8.2	Pre-exercise: 3.3 ± 1.8 Short-term post-exercise: 1.2 ± 1.6	Pre-exercise: 14.5 ± 5.0 Short-term post-exercise: 24.1 ± 4.0
	Resistant training (25–45 min per day, 3–5 times per week for 12 weeks)	T2DM	N = 11, 55.2 ± 8.2	Pre-exercise: 6.1 ± 5.3 Short-term post-exercise: 1.0 ± 0.6	Pre-exercise: 13.7 ± 5.8 Short-term post-exercise: 25.7 ± 12.1
	Combination aerobic and resistance training (25–45 min per day, 3–5 times per week for 12 weeks)	T2DM	N = 11, 55.2 ± 8.2	Pre-exercise: 7.9 ± 6.1 Short-term post-exercise: 1.3 ± 0.9	Pre-exercise: 20.7 ± 17.4 Short-term post-exercise: 30.4 ± 15.9
Kang, 2016 [24]	Combination aerobic and resistance training for 60 min per day, three times per week for 12 weeks	T2DM	N = 8, 56.0 ± 7.4	Pre-exercise: 1.12 ± 0.3 Short-term post-exercise: 1.1 ± 0.2	Pre-exercise: 17.9 ± 5.4 Short-term post-exercise: 19.9 ± 6.8
Loimaala, 2003 [25]	Endurance training twice a week and supervised muscle strength training twice a week for 12 months	T2DM	N = 24, 53.6 ± 6.2	Pre-exercise: 0.32 ± 0.16 Long-term post-exercise: 0.39 ± 0.24	Not available
Figueroa, 2007 [30]	16 weeks of walking training	T2DM	N = 8, 50.0 ± 1.0	Pre-exercise: 1.01 ± 0.06 Long-term post-exercise: 0.93 ± 0.05	Not available
Pagkalos, 2008 [31]	An aerobic exercise training program three times a week for 6 months	T2DM with CAN	N = 17, 56.2 ± 5.8	Pre-exercise: 5.0 ± 2.0 Long-term post-exercise: 4.4 ± 1.6	Pre-exercise: 13.4 ± 4.0 Long-term post-exercise: 18.1 ± 5.2
	An aerobic exercise training program three times a week for 6 months	T2DM without CAN	N = 15, 55.8 ± 5.6	Pre-exercise: 6.1 ± 3.3 Long-term post-exercise: 5.2 ± 2.4	Pre-exercise: 19.7 ± 5.4 Long-term post-exercise: 22.7 ± 7.4

In the present study, we categorized these articles into three groups: immediate effects of exercise within 60 min, short-term effects spanning 2–3 months, and long-term effects exceeding 4 months. The order of suggestion was based on the interval from baseline to post-exercise, with immediate (9 subgroups), short-term (4 subgroups), and long-term (4 subgroups) effects. Abbreviation: CAN, cardiac autonomic neuropathy; LF/HF, the ratio between the absolute power of the high-frequency (HF) band (0.015–0.400 Hz) and the absolute power of the low-frequency (LF) band (0.040–0.150 Hz); HIIE, high-intensity interval exercise (consisted of 1 min exercise bouts at 90% of VO_2 Reserve_, alternated with 1 min active recovery bouts at 60% of VO_2 Reserve_ (1:1 ratio); MICE, moderate continuous exercise (protocol intensity was set at 60% of VO_2 Reserve_, and the duration was adjusted for each participant to achieve the targeted energy expenditure) [21]; RMSSD, the root mean square of successive heartbeat interval differences.

**Table 2:** The significant factors associated with RMSSD and the LF/HF ratio by linear meta-regression.

Outcome	Variables	Estimate (95% C.I.)	S.E.	z Value	*p* Value
RMSSD					
	Mean age in years	2.36 (1.01–3.72)	0.68	3.43	0.001
	Male (%)	13.76 (3.59–23.94)	5.19	2.65	0.008
	The duration of exercise in months	1.50 (0.41–2.59)	0.56	2.69	0.007
	Intercept	−140.5 (−218.6–62.3)	39.89	−3.52	<0.001
LF/HF ratio					
	Mean age in years	0.05 (0.001–0.098)	0.02	1.98	0.048
	Intercept	−2.99 (−5.86–−0.119)	1.47	−2.04	0.041

Abbreviations: LF/HF, the ratio between the absolute power of the high-frequency (HF) band (0.015–0.400 Hz) and the absolute power of the low-frequency (LF) band (0.040–0.150 Hz); RMSSD, the root mean square of successive heart beat interval differences.

**Table 3:** The summary of different groups by immediate, short-term, and long-term effects of exercise on autonomic nervous system activity.

The Groups	Study	Types of Exercises	HRV Measurement	Influence of Autonomic Nervous System Activity after Exercise
Immediate (exercise within 60 min)				
	Marôco, 2023 [21]	MICE and HIIE	LF/HF ratio	Mild increase in the LF/HF ratio in MICE
	Bhati, 2019 [22]	A maximal exercise test	RMSSD and LF/HF ratio	Decrease in both RMSSD and LF/HF ratio
	Colberg, 2014 [28]	Walk on a treadmill and play table tennis	RMSSD and LF/HF ratio	The RMSSD and LF/HF ratio decrease during treadmill walking, while the LF/HF ratio increases and the RMSSD decreases during table tennis
	Grieco, 2014 [29]	Yoga	RMSSD	No change
Short-term (exercise between 2–3 months)				
	Piralaiy, 2021 [23]	Aerobic training, resistance training, and combination	RMSSD and LF/HF ratio	Increase in RMSSD and decrease in LF/HF ratio
	Kang, 2016 [24]	Combination aerobic and resistance training	RMSSD and LF/HF ratio	Increase in RMSSD and no change in LF/HF ratio
Long-term (exercise exceeding 4 months)				
	Loimaala, 2003 [25]	Endurance training	LF/HF ratio	Increase in LF/HF ratio
	Figueroa, 2007 [30]	Walking training	LF/HF ratio	Decrease in LF/HF ratio
	Pagkalos, 2008 [31]	An aerobic exercise training program	RMSSD and LF/HF ratio	Increase in RMSSD and decrease in LF/HF ratio

Abbreviation: HIIE, high-intensity interval exercise (consisted of 1 min exercise bouts at 90% of VO_2 Reserve_, alternated with 1 min active recovery bouts at 60% of VO_2 Reserve_ (1:1 ratio); MICE, moderate continuous exercise (protocol intensity was set at 60% of VO_2 Reserve_, and the duration was adjusted for each participant to achieve the targeted energy expenditure) [21].

## Data Availability

No new data were created or analyzed in this study.

## Supplementary Materials

The following supporting information can be downloaded at: https://www.mdpi.com/2227-9032/12/12/1236/s1, Table S1: data extraction.
