# Supplementary material for: Exercise Effects on Autonomic Nervous System Activity in Type 2 Diabetes Mellitus Patients over Time: A Meta-Regression Study
Source: Healthcare (Basel). 2024 Jun 20;12(12):1236. doi: 10.3390/healthcare12121236 (PMC11487405; doi:10.3390/healthcare12121236)
Supplement: Supplementary file 1 [file healthcare-12-01236-s001.zip › Supplementary Table S1_data extraction_b_.pdf]

Supplementary Table S1: data extraction

Publication	Title	HRV measurement	Page
Marôco, 2023 [22]	Post-acute exercise cardiovagal modulation in older male adults with and without type 2 diabetes	LF/HF ratio	Table 3
Bhati, 2019 [23]	Diagnostic performance of resting and post-exercise heart rate variability for detecting cardiac autonomic neuropathy in type 2 diabetes mellitus	RMSSD, LF/HF ratio	58
Colberg, 2014 [29]	Exercise Effects on Postprandial Glycemia, Mood, and Sympathovagal Balance in Type 2 Diabetes	RMSSD, LF/HF ratio	264
Grieco, 2014 [30]	Acute effect of breathing exercises on heart rate variability in type 2 diabetes: a pilot study	RMSSD	644
Piralaiy, 2021 [24]	Cardiac Autonomic Modulation in Response to Three Types of Exercise in Patients with Type 2 Diabetic Neuropathy	RMSSD, LF/HF ratio	1475
Kang, 2016 [25]	Effects of 12 weeks combined aerobic and resistance exercise on heart rate variability in type 2 diabetes mellitus patients	RMSSD, LF/HF ratio	2092
Loimaala, 2003 [26]	Exercise Training Improves Baroreflex Sensitivity in Type 2 Diabetes	LF/HF ratio	1839
Figueroa, 2007 [31]	Endurance training improves post-exercise cardiac autonomic modulation in obese women with and without type 2 diabetes	LF/HF ratio	440
Pagkalos, 2008 [32]	Heart rate variability modifications following exercise training in type 2 diabetic patients with definite cardiac autonomic neuropathy	RMSSD, LF/HF ratio	50
